# Buffalo Milk as a Source of Probiotic Functional Products

**DOI:** 10.3390/microorganisms9112303

**Published:** 2021-11-05

**Authors:** Márcio Vargas-Ramella, Mirian Pateiro, Aristide Maggiolino, Michele Faccia, Daniel Franco, Pasquale De Palo, José M. Lorenzo

**Affiliations:** 1 Centro de Educação Superior da Região Sul—CERES da Universidade do Estado de Santa Catarina—UDESC, Laguna 88790-000, Brazil; marcio.ramella@hotmail.com; 2 Centro Tecnológico de la Carne de Galicia, Rúa Galicia No. 4, Parque Tecnológico de Galicia, San Cibrao das Viñas, 32900 Ourense, Spain; mirianpateiro@ceteca.net (M.P.); danielfranco@ceteca.net (D.F.); 3 Department of Veterinary Medicine, University of Bari A. Moro, Valenzano, 70010 Bari, Italy; aristide.maggiolino@uniba.it (A.M.); pasquale.depalo@uniba.it (P.D.P.); 4 Department of Soil, Plant and Food Sciences, University of Bari, Via Amendola 165/A, 70126 Bari, Italy; michele.faccia@uniba.it; 5 Área de Tecnología de los Alimentos, Facultad de Ciencias de Ourense, Universidad de Vigo, 32004 Ourense, Spain

**Keywords:** functional foods, healthy dairy products, intestinal microbiota lactic acid bacteria, mozzarella

## Abstract

In the past two decades, consumption of food has been accruing due to its health claims which include gastrointestinal health, improved immunity, and well-being. Currently, the dairy industry is the sector where probiotics are most widely used, especially in fermented milk, cheese, yoghurt, butter, and dairy beverages. Although, it is still necessary to face many challenges regarding their stability and functionality in food. Considering the increasing demand for healthy products, it is necessary to develop strategies that aim to increase the consumption of functional foods in order to meet probiotic usefulness criteria and the consumer market. This review aimed to promote the utilization of buffalo milk considering its probiotic effects as a functional food and natural remedy to various ailments, emphasizing the potential of innovation and the importance of milk-based products as health promoters. The intake of probiotics plays an important role in modulating the health of the host, as a result of a balanced intestinal microbiota, reducing the risk of development of various diseases such as cancer, colitis, lactose intolerance, heart diseases, and obesity, among other disorders. However, further studies should be carried out to deepen the knowledge on the relationship between raw buffalo milk, its dairy products microbiota and consumer’s health beneficial effects, as well as to implement a strategy to increase the variety and availability of its products as a functional food in the market.

## 1. Introduction

Domesticated buffaloes (*Bubalus bubalis*) are highly adaptable and multipurpose animals [1]. Buffalo rearing has been facilitated for its versatility, efficient use of low-quality and high-fiber diets [2], parasite resistance, good quality meat, and rich milk and milk products [3]. In some countries, buffaloes have a social and cultural importance, providing draught power and an affordable source of meat and milk [4,5].

World milk production has almost doubled in the last twenty years, highlighting buffalo milk with an annual growth rate higher (~2.5%) than that presented by cow’s milk [6]. During this time, the breeds for milk production have been selected and notably improved [3]. The largest buffalo milk producers are India, Pakistan, and China, while the higher producer in Europe is Italy, ranked at the 6th position in the worldwide context [7]. Although, it is also produced in countries such as Bulgaria, Germany, or Greece [8]. Moreover, in 2019, buffalo milk was ranked at second after cow milk, representing 15% of the total worldwide milk production (~910 million tons) [6]. Fresh buffalo milk accounts for over 50% of drinking milk in India, Pakistan, Egypt, and Nepal. In addition, buffalo dairy products are traditional from Asian and Caucasian countries [9], in which dairy foods such as dahi, ghee, and yoghurt are very consumed. Despite the fact that it is not common in Europe, this type of milk has an important market in some Mediterranean countries, and the dairy buffalo industry is thriving on the gained popularity of mozzarella cheese [4,9,10,11]. In addition, the nutritional benefits of buffalo milk make it a potential substitute for cow’s milk, associated with significant milk allergies [12]. In this regard, some varieties of cheese that were made from cow’s milk are now made from buffalo’s milk [13]. This emerging global market offers a huge boost for the buffalo milk industry [14].

Milk is considered a very important source of nutrients due to the beneficial effects of many bioactive compounds present in its composition [4]. In line with this, buffalo milk presents specific properties and health-promoting characteristics for consumers, compared with other species [4,11]. The flavour, taste, and texture of buffalo milk products can be influenced by the microbial flora present in raw milk, as well as potential to contribute to health through the probiotic-associated traits [11]. Health-promoting bacteria isolated from milk and its products are commonly referred to as “probiotics”. According to the latest definition of the International Scientific Association for Probiotics and Prebiotics (ISAPP), probiotics are “live microorganisms that, when administered in adequate amounts, confer a health benefit on the host” [15]. In the last five years, the popularity of probiotics boosted substantially, and there are many experimental and clinical pieces of evidence that demonstrate their benefits for health [16]. The prevention and treatment of diseases, as well as health “optimization”, are among their biological effects [16]. In addition to dairy products, they can be consumed as raw vegetables and fruits or fermented pickles [17].

Functional foods contain bioactive compounds associated with the prevention, control, or treatment of chronic diseases [18]. Carotenoids, dietary fibers, fatty acids, minerals, prebiotics, probiotics, symbiotics, and vitamins are among the most commercialized products in the functional food market [18]. Concerning this, there is a certainty that the consumption of probiotic dairy products leads to health benefits such as the reduction of blood cholesterol, and prevention of obesity, diabetes, cardiovascular diseases, and cerebral stroke [17]. For instance, beneficial effects of yoghurt cultures like *Lactobacillus delbrueckii* subsp. *bulgaricus* and *Streptococcus salivarius* subsp. *thermophilus* have already been widely accepted [15].

However, although milks from animal species such as buffaloes are essential to the human diet in several world areas, the most part of the studies have focused on cow milk. In addition, although there are many studies about the effect that the consumption of probiotics (freeze-dried or powdered pills) has on chronic health conditions, there is hardly any research about probiotics from buffalo milk. Therefore, this review focused on studies that had evaluated buffalo milk characteristics and its products as well as the potential health benefits of the most common microorganisms isolated from raw buffalo milk. The aim of the work is to promote commercially feasible applications of buffalo milk as a probiotic functional food to health-related problems. 

## 2. Buffalo Milk Characteristics and Its Dairy Products

It is noteworthy that buffalo milk is richer than cow milk in all major constituents [2,3]. In this regard, its high energy and nutritional value is due to the fact that fat constitutes the main fraction [3,4]. Buffalo milk is also characterized by high levels of lactose, protein, and ash (Table 1). However, wide variations have been recorded with regards to buffalo milk composition. Several studies have monitored compositional changes over the years, finding that they are affected by a combination of environmental, nutritional, and genetic factors [1,2,3,4,10,19,20].

Compared to bovine milk, the high content of fat and protein [9] makes buffalo milk a very good raw material for processing, especially cheese making [21]. In relation to lipids, the fat globule size of buffalo milk is larger than other ruminants such as cows, goats, and sheep, which could affect the properties of dairy products [3,22]. On the other hand, although buffalo milk has more fat, its cholesterol content is slightly lower than cow’s milk. This could be due to the difference in the size of the fat globules [3,9].

Concerning the fatty acid composition, buffalo milk contains higher total saturated fatty acids (SFA) and lower monounsaturated fatty acids (MUFA) than cow’s milk fat, under similar conditions. The milk of buffaloes also has more conjugated linoleic acid (CLA) [4,22] and its precursors (C18:1*n*-7 *trans* and C18:2*n*-6 *cis*) [22]. However, despite the differences in the fatty acid composition among these two species, the atherogenic index was almost the same [3].

The major proteins of buffalo milk are αs1-casein, αs2-casein, β-casein, κ-casein, β-lactoglobulin, and α-lactalbumin. Unlike cow milk, buffalo milk had higher contents of αs2-casein and κ-casein (~2-fold) [22]. In addition, the higher contents of κ-casein favour the production of cheese, since they allow to accelerate the enzymatic phase of the coagulation of the rennet, and reduce the necessary amounts of chymosin [3,22]. A further difference lays in the average size of the casein micelles, which is larger in buffalo milk [3,22], and in the higher activity of some enzymes such as lactoperoxidase, alkaline phosphatase, and lactoferrin [3,27]. The plasmin activity has been studied in connection with the formation of γ-caseins from hydrolysis of β-casein; even though it is not considered to be very different from that in bovine milk, the formation of plasmin-derived fragments of β-casein has been proposed as a tool to assess milk freshness [28]. As the proteome of buffalo milk is only slightly different from that of cow, there is an antibody cross-reactivity between both types of milk. However, Sheehan and Phipatanakul [12] reported a case of a patient with cow milk allergies capable of tolerating buffalo milk. It was also previously reported that the concentration of all free amino acids, which can easily be absorbed, is higher in buffalo milk than in other milks. Besides, participating in the protein synthesis, amino acids could also have a significant effect on sensory attributes of milk, especially glutamic acid, for the umami taste in cheese [22]. Buffalo milk is often richer in lactose than cow, goat, sheep and camel milk, which would result in a good source of energy for the brain and hormonal regulation [2].

Regarding minerals, buffalo milk is high in calcium (1.5-fold higher than cow’s milk) [3,22]. The mineral content of buffalo milk (higher calcium than phosphorus content) may be a matter of concern, since there is a strong correlation between fresh cheese yield and rennet coagulation time concerning these minerals [22]. In addition, buffalo milk is considered a better supplement for infants than bovine milk due to its calcium contents, a better calcium/phosphorus ratio, and a greater protein efficiency ratio [20]. Therefore, buffalo milk has many advantages compared to other species.

Furthermore, milk from buffalo contains more tocopherols, vitamin A [9], and δ-valerobetaine (antioxidant and anti-inflammatory actions) [4] compared to bovine, as well as biliverdin, bioactive pentasaccharides, and gangliosides, which do not appear in the composition of cow’s milk [3]. Finally, volatile composition of buffalo milk shows that 50% of the identified compounds are esters, 14% aldehydes, 13% nitrogen compounds, 9% ketones, 5% aliphatic alcohols, 2.5% aromatic, and 4% sulfur compounds [3]. According to Moio et al. [29], the aroma of water buffalo milk was highly connected to l-octen-3-ol (raw mushrooms), indole (stable, animals), nonanal (freshly cut grass), and with an unidentified compound associated with a typical odour of warm milk and/or smoked cheese.

## 3. Probiotics

The word “probiotic” comes from Greek, and it means “for life” [17]. The initial notion of probiotic microorganisms dates back to a century ago when the Nobel Laureate Elie Metchnikoff suggested in his book “The Prolongation of Life” [30] that the consumption of fermenting bacilli (*Lactobacillus bulgaricus*) from milk products could positively influence the microbiota of the gut [31,32]. 

The term “probiotic” was probably invented in 1954 by Ferdinand Vergin in his article entitled “Anti-und Probiotika” [33]. This definition has been modified a lot since then. Nowadays, as already discussed, the most recent definition was formulated from FAO and WHO guidelines [34] and published in 2013 by ISAPP [15]. The most common genera currently used that possess probiotic characteristics are the lactic acid bacteria *Bifidobacterium* and *Lactobacillus* [18,32]. Table 2 shows the main microorganisms (bacteria and some yeast) that have been utilized as probiotic cultures.

### 3.1. Probiotic Strains Required Properties

The formula of probiotic cultures must be adequate to achieve in sufficient and viable number the target in the host after processing, storage, and gastrointestinal transit [16,35]. In addition to this, it is important to consider the type of carrier/matrix and the interaction between probiotic and starter culture, since it could condition the viability of a particular strain, resulting in a modification of product properties [36,37].

Taking this into account, probiotic strains must meet safety, functional, and technological usefulness criteria with documented pro-health effects consistent with the strain. The safety of a strain is related to the absence of pathogenic cultures and resistance to antibiotics, while its functional properties are related to its survival in the gastrointestinal tract and its immunomodulatory effect. Lastly, technology usefulness means that they must maintain their properties during storage and distribution [17].

In addition to the abovementioned aspect, probiotics are also subject to food regulations. For example, in the USA, the FDA (Food and Drug Administration) grants the GRAS (Generally Regarded As Safe) status to all microorganisms used in food [17]. In Europe, the EFSA utilizes the QPS term (Qualified Presumption of Safety) [38], defined as “an assumption based on reasonable evidence”, which allows certain restrictions to apply [39]. This qualification is awarded when a group of microorganisms does not pose safety concerns [40]. In this regard, the EFSA has awarded this qualification only to 32 *Lactobacillus* species for human applications.

**Figure 1:**
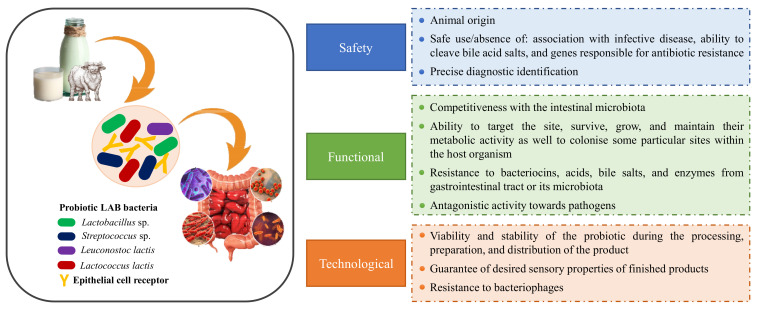
Selection criteria that should be applied to raw buffalo milk and its products to consider its microbiota as probiotic strains concerning its safety, functional, and technological properties. Adapted from [34,43].

Nowadays, the most studied and employed probiotic microorganisms with proven efficacy for human health are *Bifidobacterium animalis*, *Lactobacillus casei* Shirota, *Lactobacillus rhamnosus* GG, *Saccharomyces cerevisiae* Boulardii, and as well *geni Enterococcus*, *Lactococus*, and *Streptococcus* [17,35,41]. These strains have long been used safely in the food industry [42]. On the other hand, recent advances have been made in the selection and characteristics of new probiotic strains, their possible use, and their health effect [17]. In Figure 1 is shown the selection criteria that should be applied to raw buffalo milk and its products to consider its microbiota as probiotic strains according to the FAO [34] and EFSA [43] concerning its safety, functional, and technological properties.

Lastly, there is growing concern about the misuse of the term “probiotic”, used in many products without meeting the required criteria. This is the case of products such as aftershave, disinfectants, mattresses, or shampoos, where the viability and efficacy of the microbes used has not been taken into account [15]. In addition, sometimes it is difficult to know the contribution of these microorganisms to human health, since sometimes these microorganisms are not well defined in foods and could fall short to be considered probiotics. Therefore, it is recommended to use the statement “contain live and active cultures”, and not to misuse the term probiotic [15].

Therefore, as mentioned above, the growing interest in probiotic products has motivated regulatory bodies to protect consumers from the misuse of nutrition and health claims on food. Nevertheless, sometimes a sufficient level of scientific evidence is not reached by agencies responsible for securing health benefits, which makes this process not standardized throughout the world [18]. In this regard, no claims on probiotics are listed on the European Union register as authorised for use.

### 3.2. Mechanism of Action and Human Health Effects of Probiotics

Three mechanisms are responsible for the promotion of human health by probiotics: (i) provide end-products of anaerobic carbohydrate fermentation to the host as well as affect other microbial products such as host products (e.g., bile salts), food ingredients, and toxins; (ii) direct effect on other microorganisms (commensal and/or pathogenic); and (iii) stimulate host immune response (innate and acquired), which is so important in the prevention and treatment of diseases and the eradication of neoplastic host cells. It is important to mention that the health effects of probiotics are not specific to the species or genus, but rather strain-specific [18,35,44]. In this case, microorganisms with probiotic effect are unlikely to be a universal remedy for various diseases, since they do not respond at the same time to the three mechanisms of action mentioned above. In this regard, probiotic products can be made up of one or more microbial strains. This selected combination of probiotics would offer a higher level of protection, since it would show several mechanisms of action at the same time.

Despite the fact that, at this time, a consensus has not been established on what is the adequate intake of probiotics [16], the particular advantage of probiotics is their mechanisms of action. Probiotics efficacy is directly associated with their effect on other microorganisms (e.g., pathogens) and the balance of the host’s intestinal microbiota. Their ability could promote the formation of a protective barrier, which would prevent the colonization of the epithelium by pathogenic bacteria, protecting the host from toxins as well as causing the activation of immune cells [17,35]. Immunological stimulation of probiotics can consist in an increasing production of immunoglobulins and γ-interferon, macrophages and lymphocytes activity. In addition, the components of the cell wall of lactic acid bacteria (LAB) can encourage the activity of macrophages, which would increase the production of free oxygen radicals and lysosomal enzymes, destroying microbes rapidly [17]. Thus, as already discussed, the consideration of a microorganism as a probiotic depends mainly on the ability of a strain to survive through the GI tract and colonise the intestine.

At the same time, there are several techniques to maintain the viability of these microorganisms in the intestinal system. Among them, the addition of growth promoters (e.g., prebiotics) or the use of microencapsulation techniques stand out [18]. Prebiotics are described as a non-viable food component that modulates the microbiota in order to give a health benefit to the host [45]. There are many studies that demonstrate the beneficial effects of prebiotics, highlighting the results achieved with the combined use of probiotics and prebiotics. The resulting food is known as “synbiotic", whose purpose is to improve the survival of probiotic microorganisms in the GI tract. The most promising prebiotic substances are oligosaccharides such as fructooligosaccharides, galactooligosaccharides, isomaltooligosaccharides, xylooligosaccharides, transgalactooligosaccharides, and soybean oligosaccharides [17,35].

On the other hand, concerning probiotic encapsulation, the most common methods are extrusion, emulsion, coacervation, lyophilization, and spray drying. Probiotics encapsulation can promote the viability and functionality of microorganisms in food products. The microencapsulation technique is presented as a great alternative to maintain probiotic viability during food processing and its passage through the gastrointestinal tract. In addition, this technique can mask unpleasant flavours and odours, thus increasing the product shelf life [31].

In contrast, the disadvantages arising from the use of probiotics have sparked interest in the development of non-viable probiotic preparations. There is a growing conviction that the benefits attributed to physiologically active bacteria are not strictly associated with their viability. Recent studies have suggested to this preparation terms such as paraprobiotic (from “probiotic paradox”), non-viable, inactivated, tyndallized or ghost probiotics. Paraprobiotics are defined as non-viable microbial cells or raw cell extracts that could lead to health benefits when consumed in adequate amounts [16].

### 3.3. Probiotic Microorganisms of Buffalo Milk

Raw milk can contain a diverse bacterial population. In addition, its high nutrient content with a high-water activity at a near-neutral pH promotes the growth of many microorganisms. However, despite the fact that the microbial community within milk is complex, many raw milk isolates can contribute to the technological properties of dairy products and its health-promoting abilities [11]. This specific microbiota is going to condition the development of dairy products and, among probiotics, dairy products are the key product sector [42]. LAB (e.g., *Lactococcus*, *Streptococcus*, *Pediococcus*, and *Enterococcus*) are the main probiotic group, while lactobacilli and bifidobacteria (usually found as commensals in the human gastrointestinal tract) comprise the most used and recognized probiotic microorganisms [18,31].

**Figure 2:**
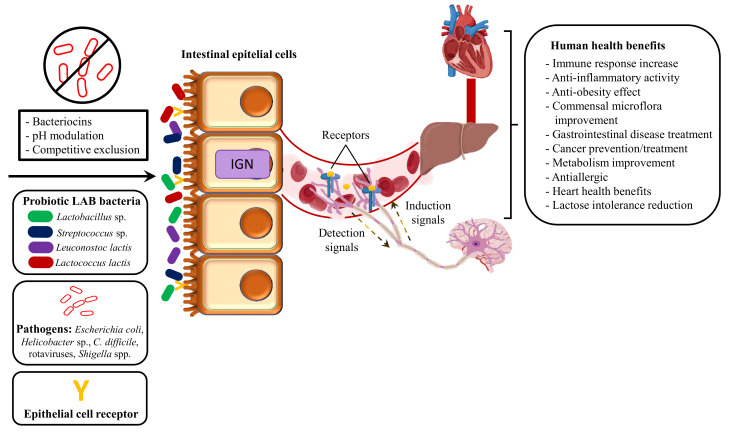
Mechanism of action and human health benefits from the main lactic acid bacteria (LAB) isolated from raw buffalo milk (*Lactobacillus* sp.: *L. plantarum*, *L. paracasei*, *L. fermentum*, *L. delbrueckii*; *Streptococcus* sp.: *S. thermophilus*, *S. macedonicus*).

Concerning buffalo milk, it is generally accepted that, prior to pasteurization, LAB species are the dominant bacterial population. *Lactobacillus*, *Lactococcus*, *Streptococcus*, *Leuconostoc*, and *Enterococcus* are the most common LAB in buffalo milk [11]. The microbial content less prevalent but frequently isolated from raw buffalo milk also includes psychrotrophic populations (*Pseudomonas* and *Acinetobacter* spp.), which particularly establish themselves during cold storage, *Staphylococcus* species, and *Escherichia coli* [11]. Nevertheless, some probiotics create uncertainty about their safety. This is the case of the genus Enterococcus, which can be pathogenic and cause disease in the host [32]. The potential human health benefits as well the mechanism of action of the most common LAB probiotics isolated in raw buffalo milk are shown in Figure 2.

The main beneficial effect of probiotics is to repair the gut microbiota, which allows to improve gut and immune homeostasis. Along with these, probiotics can have modulatory effects on CNS disorders (anxiety, depression, and ASD) through modifications in the gut microflora composition [46]. In this regard, several studies have demonstrated that gut microbiota has a key role in the gut–brain axis. This bidirectional interaction between microbiota and brain receives the name of microbiota/gut–brain axis.

The most studied microorganisms isolated from raw buffalo milk with probiotic and/or technological effects in dairy products (*Lactobacillus* sp., *Streptococcus* sp., *Lactococcus lactis*, *Leuconostoc lactis*, and *Pseudomonas fragi*) are discussed in more detail below.

#### 3.3.1.* Lactobacillus*

There are several isolates of dairy lactobacilli bacteria that have demonstrated effective as probiotic strains. The inhibition of pathogenic organisms, the reduction of lactose intolerances and the increase of the immune response are associated with probiotics of the genus *Lactobacillus* [11]. *Lactobacillus* genera can produce bacteriocins and some antibiotics, such as the so-called de-conjugated bile acids, which display higher antibacterial effects than those shown by bile salts produced by their host [44]. The main *Lactobacillus* species with probiotics properties isolated from buffalo milk are *L. plantarum*, *L. paracasei*, *L. fermentum*, *L. delbrueckii*, and *L. kefiranofaciens* [11].

The genus *Lactobacillus* exhibits a level of genetic diversity superior to that found in other bacterial genera. Since 2015, several large-scale phylogenetic analyses, based on the phylogeny of the central genome, have revealed the discrepancy between these taxonomies. Therefore, the taxonomy of *Lactobacillus* has been changed to create a genus with a better homogeneity between organisms. The new classification, based on various approaches and genetic markers [47], provides a better ecological and functional vision that even can improve the way the industry develops products.

*L. plantarum* (now known as *Lactiplantibacillus plantarum*) is a natural producer of bacteriocins and B group vitamins [48]. The production of bacteriocins from this microorganism could improve the safety and increase the quality of dairy products [44]. *L. plantarum* also enhances the absorption of vitamins and minerals as well as stimulates the generation of organic acids and amino acids. In addition, these probiotic increases provide protection against pathogens (e.g., blocking adherence of pathogens or by mucin production) and the efficiency of the immune system [17,44]. It was previously reported that *L. plantarum* competes with pathogens (e.g., *Helicobacter* sp., *Clostridium difficile*, rotaviruses, *Shigella* spp., and *E. coli*) for receptor sites in the GI tract more effectively than that achieved by other species. In addition, reduction in luminal pH, competition for nutrients, and production of beneficial volatile compounds are the main mechanisms responsible for the competitive exclusion of pathogens [49]. Finally, teichoic acid from the cell wall of *L. plantarum* could be related to its anti-inflammatory activity [44].

It was previously reported that *L. paracasei* consumption had anti-obesity effects with body weight, body fat, and feed efficiency decrease, probably due to the reduction of the average radius and the consequent increase in the number of small adipocytes [50]. In addition, *L. paracasei* could improve immune function by enhancing the systemic immune response to challenges, like vaccinations in healthy subjects. Particularly, previous studies suggested that daily consumption of yoghurt containing *L. paracasei* could improve immune function by enhancing NK cell activity [51]. Increased concentrations of innate and acquired immunity biomarkers have been reported after the use of *L. paracasei*, with short-chain fatty acids and volatile compounds (e.g., faecal butyrate) modulation, and were also reported as beneficial effects of this probiotic against diarrhea and constipation [49].

Nowadays, there is a growing interest in the health benefits of polyphenols (e.g., ferulic acid), since several properties are associated with them such as antidepressant, anti-inflammatory, neuroprotective, antihyperglycemic, anticancer, and antidiarrheal properties [52]. However, natural sources of ferulic acid are not readily bioavailable and must be released enzymatically to be absorbed by human digest. *L. fermentum* (now known as *Limosilactobacillus fermentum*) is characterized by a high ferulic acid esterase activity [53]. Therefore, orally ingested microencapsulated ferulic acid esterase produced by *Lactobacillus* could increase bioavailability of ferulic acid [53].

Iron is considered a limited substance in the host. In fact, excluding lactobacilli, it is a crucial compound for many bacteria. *L. delbrueckii* conditions the activity of other microorganisms by binding iron hydroxide to its surface, which would make it no longer available to other pathogenic microorganisms. This gives this probiotic a very important advantage over other microorganisms that depend on this mineral [44]. *L. delbrueckii* can become established in the gut and progressively control the natural microflora. This microorganism is also able to resist acidity and reach the intestine in a viable state, which would avoid the presence of pathogens microorganisms, protecting the intestinal health of the host [54].

*L. kefiranofaciens* is one of the microorganisms that dominates the microflora of kefir and kefir grains, being able to produce kefiran. Previous studies published that *L. kefiranofaciens* is highly stress-tolerant (during industrial processes and digestive transit), to heat (52 °C), cold (−20 °C), acid (pH 3.0), and bile salts (0.2%) [55]. In addition, it was also reported that *L. kefiranofaciens* inhibits the growth and formation of biofilms against microbes, which causes dental caries, as well as palliate the enterohaemorrhagic action of *E. coli* (EHEC) infection, bacterial translocation, and intestinal and renal dysfunction [56].

#### 3.3.2. *Streptococcus*

*Streptococcus thermophilus* is the second most important specie of industrial LAB after *Lactococcus lactis*. This probiotic has GRAS status and is extensively used for the fermented dairy foods processing. *S. thermophilus* has several functional activities such as the production of extracellular polysaccharides, bacteriocins, and vitamins, as well as several health effects, transient survival, and moderate adherence in the GI tract [57]. *S. thermophilus* has similar mechanisms of action compared with those already discussed for *L. delbrueckii*. This microorganism can also establish and dominate the natural microflora of the intestine of the host due to its resistance to acidity, protecting the intestinal health of the consumer [54]. In addition, the *S. thermophilus* strain generates large amounts of folate, necessary in DNA repair of epithelial cells [44]. *S. thermophilus* can signal an immune regulatory processes and improved mucosal barrier injury, reducing the levels of pro-inflammatory factors and cytokines as well as neutrophil infiltration [49].

The recently described *Streptococcus macedonicus* is among the microorganisms similar to *Streptococcus thermophilus*. This dairy streptococcus possesses a food grade and has no potential pathogenicity. Certain strains of *S. macedonicus* produce bacteriocins named macedocin (a food-grade lantibiotic) and exopolysaccharides. However, *S. macedonicus* does not survive the low pH conditions of the stomach [58].

#### 3.3.3. *Lactococcus*, *Leuconostoc* and *Pseudomonas*

From *Lactococcus* genera, *Lc. lactis* is the most important specie. This bacteria is considered to be the most important specie of industrial LAB [57]. Fermented milk with *Lc. lactis* was previously reported as providing heart health benefits in vitro, attenuating blood pressure, cholesterol (LDL), and triglycerides levels. Hence, authors concluded that daily consumption of fermented milk with *Lc. lactis* could be used as a potential functional food [59]. In addition, *Lc. lactis* is able to survive in low pH conditions, bile salts, and self-aggregating and co-aggregating with *E. coli*, making this microorganism a potential probiotic starter culture [60].

Concerning *Leuconostoc lactis*, a previous study suggested that this microorganism would allow to produce a new and safe functional product by hydrolysing milk casein during fermentation. This probiotic showed the ability to develop an active peptide with antioxidant [61,62] and angiotensin-I converting enzyme inhibitory activity (ACE-I). The active peptide (MVPYPQR) produced during fermentation could be used as an ingredient in nutraceuticals and functional foods [61].

*Pseudomonas* species are the most commonly found psychrotrophs in chilled raw milk because of inadequately disinfected milking equipment [63]. *Pseudomonas fragi* is recognised as a common agent that contributes to the spoilage of pasteurised and UHT milk through the activity of thermostable extracellular proteases and lipases. In consequence, this microorganism can limit the shelf life of milk and its products (e.g., cheese) because of fruity off-odours as a result of the production of methyl esters, in particular, short-chain ethyl esters. In addition, some *P. fragi* isolates also produce extensive amounts of volatile compounds such as methyl and ethyl acetate, ketones, alcohols, 1-undecene, and sulphur compounds [63,64]. In order to maintain the quality of the dairy products, controlling the growth of *Pseudomonas* and their activity in milk prior to heat treatments is of great importance [64,65].

## 4. Functional Food: Milk-Based Products from Buffaloes

The diet provides the necessary nutrients to meet metabolic needs. However, growing concern for health and well-being is increasing the demand for functional foods, which has motivated the change in the way of conceiving food [35]. This is also reflected in the interest in oral probiotics and their effects on the gut microbiota [31]. Functional food can be defined as “Similar-looking foods to conventional food intended to be part of a normal diet, which has been enhanced to offer physiological functions beyond the provision of the nutrient requirements” [42].

Originally, the functionality of functional foods was based on bioactive components that are often already part of the food. Today, probiotics, prebiotics, vitamins, and minerals are also included among these functional ingredients, which are commonly used in developing dairy products [32,35]. However, the viability of probiotics in food is a critical factor. Therefore, some food matrices are more suitable to deliver probiotics than others. The buffering capacity of milk and milk fat guarantees the survival of probiotics throughout the production process. Hence, dairy products rich in milk fat, including yoghurt (a very efficient probiotic vehicle), cheese, and frozen fermented dairy desserts, represent an ideal and marketable carrier of functional food [16,35]. To ensure the declared benefits, it is necessary that, at the time of consumption, the probiotics be viable and sufficiently abundant [66]. In addition, to have a therapeutic effect, it must contain at least 10^6^–10^7^ CFU/g of probiotic bacteria, or a total of 10^8^–10^9^ CFU, taking into account an approximate daily consumption of 100 g or 100 mL of probiotic food [31].

Buffalo milk can be used to make a wide variety of dairy products, since it contains less water and more fat, which is especially useful for the production of dairy products. In addition, buffalo milk is also ideal for the manufacture of these products due to the larger globule size and the higher proportion of solid fat [2]. In this sense, fermented dairy foods such as yoghurt and cheese have been traditionally associated with functional food, since they are the major vehicle in delivering probiotics [18,32]. In addition, fermented milk is one of the best matrices to elaborate functional foods, since it has a high sensory acceptance and a long shelf life [66]. The potential technological properties in dairy products from the most common probiotics microorganisms isolated in raw buffalo milk are shown in Table 3.

On the other hand, it is important to highlight that most of the dairy products described in this review are from pasteurized buffalo milk (or similar heat treatment) and, therefore, the probiotic microorganisms from raw milk become inactive after processing. The most studied dairy products in which probiotic microorganisms isolated in raw milk of buffalo are commonly utilized (cheese, yoghurt, kefir, butter, and dairy beverages) are further discussed below.

### 4.1. Cheese

Cheese provides a favourable environment to maintain the viability of the probiotic strains until the moment of consumption [18]. In this sense, several studies have shown the possibility of making different types of cheeses with various types of probiotic strains. This allows dairy industries to diversify their products in an increasingly competitive market.

Concerning buffalo milk used for the manufacture of dairy products, it is highly appreciated for its chemical composition that determines its nutritional properties and its suitability to produce functional products. In this regard, the high variability of triglycerides and fatty acids allows to separate fat into various fractions based on its melting characteristics. Thus, its high content of high-melting triglycerides contributes to its higher density and makes it suitable for making cheese [2].

In Italy, mozzarella cheese is usually manufactured with buffalo milk, as well as in Balkan countries where white-brined and pickled cheeses are made from this product [2]. In relation to buffalo cheese nutritional value, Becskei et al. [2] observed that lysine was the major amino acid, followed by branched amino acids (valine, isoleucine, and leucine), which promote protein synthesis and are metabolized for energy in the muscles rather than the liver. Among the non-essential amino acids, glutamic acid was the one that showed the highest content in this product.

In relation to probiotic microorganisms, buffalo mozzarella is characterized by the presence of *Lactobacillus* and *Streptococcus* genera [68]. *S. thermophilus*, *L. delbrueckii*, and *L. helveticus*, followed by *Acinetobacter* and *Pseudomonas* sp., are the predominant bacteria. On the other hand, as already discussed, improvements are needed in the production of hard cheese from buffalo milk in order to maintain the desirable viability of probiotic cultures because of the processing that includes heat treatment and mucor rennet as methods [2].

In addition, improvements are also necessary if it is intended to use probiotic bacteria such as *S. macedonicus*. For example, the optimum temperature (20–25 °C) for macedocin (a biopreservative bacteriocin) production by *S. macedonicus* is noticeably below that of growth (42 °C). In contrast, the pH reached at the start of milk fermentation for macedocin production is almost the same as that for growth (pH 6.4). Besides, macedocin is retained in the matrix since part of its activity is associated with the curd fraction (after drainage of the whey). Moreover, salt and oxidative stress enhances specific bacteriocin production [58]. Therefore, the conditions for growth and bacteriocin synthesis by *S. macedonicus* could be promising for their effective application in several cheese varieties from buffalo milk, if improvements are applied.

Finally, it is worth noting the usefulness of *S. macedonicus* as adjuvant or co-culture, since it intervenes in the hydrolysis of fats and casein, and in the consumption of citrate. Previous studies demonstrated that mixing starter cultures of *S. thermophilus* and *S. macedonicus* would be useful for designing new starter formulations [58].

### 4.2. Yoghurt

Bovine milk is the most popular raw ingredient to produce yoghurt, but also several studies have been conducted on buffalo yoghurt due to the differences between their composition and physical properties [69].

Yoghurt is made using a mixture of thermophilic starter cultures based on *L. delbrueckii* and *S. thermophilus*. Recently, other starter cultures such as *L. acidophilus* and *Bifidobacterium* spp. have been included [69]. A higher proteolytic activity was observed when using buffalo milk. This would be due to a better growth of *S. thermophilus* and *L. bulgaricus*, and a higher titratable acidity. Moreover, the yoghurt produced with buffalo milk has excellent nutritional properties, which is related to the fact that this product generally has properties similar to the milk with which it is made [20]. In this regard, the yoghurt produced with buffalo milk would be more nutritious due to its higher concentration of protein and fat [69].

In addition, the higher concentration of milk fat and calcium could positively condition the viability of *Lactobacillus* and *Bifidobacterium* by stimulating the growth of LAB [70]. This viability can also be increased using higher concentrations of lactose, which would act as a protective layer [69]. Finally, it is important to highlight that the extension of shelf life is limited in probiotic yoghurt due to the oxidative stress suffered by probiotic bacteria. In this regard, many cultured dairy products do not meet the first criterion for probiotics, e.g., “containing live microorganisms” at consumption time, because probiotic strains cannot endure the acidity, which can even increase when it contains lactic acid-producing bacteria [16].

### 4.3. Kefir

Kefir is a fermented milk produced by a group of bacteria and yeasts (e.g., with kefir grains). This microbial fermentation can be carried out in a traditional or commercial way [71]. Concerning the artisanal production, the milk is inoculated with a variable quantity of grains that are sieved at the end of the fermentation process, and they could be used for a new fermentation or kept in fresh milk. The initial inoculum concentration of the grains affects the microbiological profile of the final product [72]. Kefir has been associated with health benefits such as anti-allergenic, anti-asthmatic, anticancer, anti-microbial, anti-stress, cholesterol-lowering, immune-modulation, and gastrointestinal beneficial effects [71].

The microbial flora of kefir contains acetic acid bacteria, LAB (Acinetobacter, Enterobacter, Enterococcus, Lactobacilli, Lactococci, Leuconostocs, Pseudomonas spp. and Streptococci), and yeasts (Candida, Kluyveromyces, Rhodotorula, Saccharomyces, Torulopsis and Zygosaccharomyces). In addition, the kefir’s properties depend especially on the milk used and its origin and production method [56]. The effects of buffalo milk on kefir characteristics were previously reported by Gul et al. [71], who found higher counts of Lactococci and yeast in kefir samples made from buffalo milk than those made from cow’s milk at the end of the storage period.

### 4.4. Butter and Ghee

Butter and ghee (butter oil) are popular dairy products made from buffalo milk in several countries [9,73]. Buffalo milk produces a whiter butter than cows’ milk due to the lack of carotenoids [74], with a higher level of saturated fatty acids (harder product) and yield. In addition, it has higher stability because of the slower rate of fat hydrolysis (free fatty acids release) [2,75]. However, probiotics are most commonly used in plant-based butters such as peanut butter and butters from coconut, flaxseed, and sunflower oils. Although, recent studies have suggested the utilisation of probiotic bacteria (*Bifidobacterium bifidum*, *Lactobacillus acidophilus*, and *L. maltaramicus*) in butter elaborated from milk [76,77]. Conversely, probiotics strains usually isolated from commercially available fresh butter are not present in raw buffalo milk. On the other hand, probiotic microorganisms of buffalo milk *L. delbrueckii* and *S. thermophilus* were recently isolated from homemade butter and reported as starter cultures to butter fermentation processing [77]. To obtain the beneficial effects of butter as a functional food is recommend its consumption level at 25 g per day [76].

### 4.5. Dairy Beverages

Dairy beverages are products made by blending milk with whey, vegetable fat, fermented milk, and other products [36]. The majority of the probiotic dairy beverages can be categorized into fermented products [32]. The production of beverages based on buffalo milk would allow to expand the range of products, offering different qualities to those of products traditionally made with cow’s milk.

A previous study comparing probiotic dairy beverages between buffalo and cow products evaluated the viability of the common microorganisms used to produce fermented dairy products *S. thermophilus*, *L. bulgaricus*, and *L. acidophilus* during storage as well their resistance to in vitro gastrointestinal conditions. LAB showed viable cell counts at the end of shelf life with higher values for buffalo dairy products, which suggested the beneficial protective effect on human microbiome that this product might have compared to cow products [36].

## 5. Health Effects of Probiotics

Evidence of human health benefits in favour of probiotics has been extensively investigated [15,17,18,31,44,49,66,78]. Meanwhile, it is necessary to highlight that the probiotics benefits are strongly related to the probiotic strains. In addition to probiotics, bioactive compounds produced by fermentation would also have health benefits [66]. Taking this into account, Table 4 summarises the probiotic strains that are commonly found in raw buffalo milk and their potential health benefits if consumed as raw milk and/or dairy products as functional food.

Lastly, with the expansion of the healthcare industry globally, the market for probiotics is expected to continue to grow in the world (mainly in Asia) [31]. Also, over time, the perspective on meeting hunger needs, survival, and anticipating adverse effects has changed towards the use of foods that increase the state of well-being, improving health and reducing the risk of suffering from some diseases. It is important to attend to these aspects, especially taking into account the increase in medical care in some countries [66].

## 6. Probiotic Adverse Effects

Concerning intake of probiotics (live microorganisms), bacteraemia and fungemia are among the most frequent ailments, although this list is expected to be expanded in the near future. The main concerns include the possible transfer of genes from pathogenic bacteria in the intestine, the specific properties of the strain, and the competition with the intestinal microflora. Bearing this in mind, food safety is a relevant challenge in the production of probiotics, especially taking into account that the coming generation of probiotics will incorporate new species such as *Akkermansia muciniphila*, *Faecalibacterium*, and *Bacteroides* species [16].

In addition, the possible adverse effects of probiotics are also a matter of concern, especially those that could affect risk groups. Their consumption in high concentrations could cause immune disorders by negatively influencing the balance of cellular functions. Even if rare, cases of mortality, sepsis, endocarditis, pancreatitis, and bowel ischemia have been registered [16].

## 7. Other Functional Properties of Buffalo Milk

Milk and dairy products are a major source of bioactive peptides. These compounds are small molecules that can be present in foods and can positively modulate human health, exerting beneficial effects (e.g., anticancer, antidiabetic, antihypertensive, antimicrobial, antioxidant, and immunomodulatory properties) [9]. In this regard, peptides participate in several physiological tasks such as cardiovascular, endocrine, gastrointestinal, immune, and nervous functions. Based on the peptidomic profile of buffalo-milk dairy products, Basilicata et al. [9] found a large number of peptides with particular bioactivities and proven health benefits. Considering this, these findings would allow the isolation of new active compounds by the pharmaceutical industry, as well as the development of healthy products using buffalo milk as a functional ingredient. this functional matrix could even be combined with other compounds to achieve a synergic or additive associations.

Furthermore, the nutritional and functional value of buffalo milk can also be ascribed due to the presence of a δ-valerobetaine, short-chain acylcarnitines, and L-carnitine. These compounds can confer to the milk consumer a high antineoplastic, antioxidant, and anti-inflammatory effect [95]. In addition, buffalo milk also contains γ-butyrobetaine and L-carnitine precursors [4].

## 8. Conclusions and Future Perspectives

It is a challenge to maintain the standard limit and the viability of probiotics on functional foods (e.g., dairy products) until the end of its shelf life, as well as during the transit through the gastrointestinal tract of the consumer. Consequently, the enormous relevance currently devoted to the functional foods increased interest in maintaining the viability of probiotics and developing new types of oral delivery systems.

In this sense, microencapsulation allows to improve the viability and functionality of probiotics, and constitutes a key research niche. The microencapsulation technique is presented as a great alternative in maintaining probiotic purpose. Additionally, another challenge is using adequate methodologies to assess the efficacy of probiotic microorganisms and ensure their positive health effect. Their application must necessarily go through the correlation of the biological response with the physical condition and vitality of the microbial species.

In conclusion, this review presents the latest discussions on the probiotic microorganisms of raw buffalo milk and its milk-based products application as functional food. Numerous scientific reports confirm that probiotic organisms are key to the balance of the gut microbiota and have a positive effect on the host’s health. Besides, probiotics had an already recognized application in dairy products, which is a great opportunity for the functional food development.

In line with this, our review confirms that buffalo raw milk has several probiotic microorganisms and it could be satisfactorily used in the production of high-quality traditional dairy products, giving them added value and beneficial health characteristics. Buffalo milk has also shown better physicochemical, compositional, and nutritional attributes than cow’s milk. Furthermore, a wide dissemination of the abovementioned proposal for buffalo milk could actively contribute to the promotion of sustainable livestock exploitation, encouraging the genetic diversity and assessment of different breeds for food production.

Finally, with the aim to explore several benefits from the most important livestock that provides milk used by humans for dairy products (e.g., cow, buffalo, goat, sheep, and camel), mixing different types of milk to develop functional foods with enhanced sensorial properties as well improve their health benefits could be an interesting alternative for future research.

## Figures and Tables

**Table 1:** Buffalo (*Bubalus bubalis*) milk chemical composition and nutritional value.

Compound	Value	Reference
Milk density (g/cm^3^)	1.037	[2]
***Chemical composition***		
Dry matter (%)	16.10	[2]
Solid non-fat (%)	10.48	[2]
Protein (%)	2.70–5.20	[3,10,21,22]
Casein (g/100 mL of milk)	3.07–3.20	[19]
Fat (%)	6.02–8.80	[2,3,10,21]
Lactose (%)	4.51–5.36	[2,3,21,22]
Total ash (%)	0.60–0.90	[2,3,22]
Energy (kJ/kg)	3450–4.054	[2,21]
***Amino acids (g/100 g protein)***		
Threonine	1.99–5.71	[22,23]
Cysteine	0.59	[23]
Valine	6.76–8.28	[22,23]
Methionine	0.93–1.99	[22,23]
Isoleucine	3.32–5.71	[22,23]
Leucine	6.12–9.79	[22,23]
Tyrosine	3.36–3.86	[22,23]
Phenylalanine	3.97–4.71	[22,23]
Lysine	7.50–9.84	[23,22]
Cholesterol (mg/100 g of milk)	6.50–17.96	[3,24]
***Fatty acids (%)***		
C4:0	3.66–4.18	[25]
C6:0	1.67–2.78	[25]
C8:0	1.82–2.98	[25]
C10:0	1.80–3.21	[25]
C12:0	2.39–3.92	[25]
C14:0	9.96–10.97	[25]
C16:0	26.49–30.17	[25]
C16:1*n*-7	1.24–2.02	[25]
C18:0	10.78–13.79	[25]
C18:1*n*-9	23.33–25.17	[25]
C18:2*n*-6	1.07–1.84	[25]
SFA	63.31–68.31	[25]
MUFA	25.27–28.32	[25]
PUFA	2.43–3.10	[25]
CLA	0.41–0.58	[25]
***Minerals (mg/100 g)***		
Ca	112–148	[22,26]
P	99–107	[22,26]
K	86–92	[22,26]
Mg	8–14	[22,26]
Na	35–37	[22,26]
Zn	0.41–0.46	[22,26]
Fe	0.16	[26]
Cu	0.04	[22,26]
Mn	0.03–0.07	[22,26]

SFA: Saturated fatty acids; MUFA: Monounsaturated fatty acids; PUFA: Polyunsaturated fatty acids; CLA: Conjugated linoleic acid.

**Table 2:** Microorganisms (bacteria and some yeast) that are used as probiotics.

Genera	Species
*Lactobacillus*	*acidophilus, casei, crispatus, delbrueckii subsp. bulgaricus* ^a^ *, fermentum, gasseri, johnsonii, paracasei, plantarum, reuteri, rhamnosus, helveticus, lactis, sporogenes*
*Bifidobacterium*	*bifidum, breve, infantis, longum, lactis, animalis, adolescentis, essensis, laterosporus*
*Escherichia, Saccharomyces, Kluyveromyces, Streptococcus, Enterococcus* ^b^ *, Propionibacterium, Pediococcus, Leuconostoc, Bacillus, Clostridium*	*Escherichia coli* Nissle, *Saccharomyces boulardii, S. cerevisiae, Kluyveromyces lactis, Streptococcus thermophilus* ^a^*, S. cremoris, S. diacetylactis, S. intermedius, S. salivarius, Enterococcus francium* ^b^*, Propionibacterium freudenreichii, P. freudenreichii subsp. shermanii, P. jensenii, L. lactis, Pediococcus, Leuconostoc lactis subsp. cremoris, L. lactis subsp. lactis, Bacillus cereus, Clostridium butyricum*

^a^ poor survival during gastrointestinal transit; ^b^ potential pathogenicity and vancomycin resistance. Adapted from [18,32].

**Table 3:** Main microorganisms isolated in raw milk of buffalo (*Bubalus bubalis*) with probiotic and/or technological properties in dairy products.

Genera	Species	Technological Effects	Products	Ref.
**Most prevalent**				
*Lactobacillus*	*plantarum^(Q)^, paracasei^(Q)^, fermentum^(Q)^, delbrueckii^(Q)^, kefiranofaciens^(Q)^*	Proteolysis, lipolysis, and aroma compounds	Cheese	[11]
*Lactococcus*	*lactis^(Q)^*	* Starter cultures; flavour compounds; acidification; proteolysis; citrate utilisation; fat metabolism; bacteriocin production.	Cheese	[11]
*Streptococcus*	*thermophilus^(Q)^, macedonicus*	*Starter culture; lactose to lactate; exopolysaccharide and bacteriocins production.	Yoghurt, cheese	[11]
**Less prevalent**				
*Leuconostoc*	*lactis^(Q)^*	CO_2_ production; lactose and citrate metabolization; bacteriocins production.	-	[11]
*Pseudomonas*	*fragi*	Spoilage via heat-stable enzymes and fruity off-odour.	Milk and its products	[63,64]

* *Streptococcus thermophilus*. ^(Q)^QPS (Qualified Presumption of Safety) microorganisms [67].

**Table 4:** Potential human health benefits from probiotics microorganisms isolated from raw buffalo (*Bubalus bubalis*) milk on functional foods.

Probiotic Microorganism		Source	Health Benefits	Ref.
*Lactobacillus*	*plantarum*	Fermented milk (commercial product)	Total cholesterol, low-density lipoprotein cholesterol (LDL) and γ-glutamyl transpeptidase reduction	[79]
		Fermented soy milk	Type II diabetes (antioxidative properties and decrease of risk).	[80]
		Kefir and yoghurt	Lactose intolerance: symptoms reduction	[81]
		Yoghurt	Immune function improvement: NK cell function and IFN-γ concentration	[51]
		Encapsulated bacteria	Gut mucosal barrier improvement (post-operative treatment)	[82]
		Rose-hip drink	Irritable bowel syndrome symptoms treatment	[83]
		Cheese	Obese hypertensive treatment	[84]
		Commercial probiotic mixture	Overweight beneficial effects: HDL cholesterol, insulin sensitivity, and amelioration of inflammation (hsCRP)	[85]
	*paracasei*	Yoghurt	Immune function improvement: NK cell function and IFN-γ concentration	[51]
		Fermented soy milk	Anti-obesity	[50]
		Commercial probiotic mixture	Overweight beneficial effects: HDL cholesterol, insulin sensitivity, and amelioration of inflammation (hsCRP)	[85]
	*fermentum*	Microencapsulated probiotic	Total cholesterol, LDL cholesterol, and triglyceride reduction	[86]
		Commercial probiotic	Atopic dermatitis treatment	[87]
	*delbrueckii*	Commercial probiotic mixture	Overweight beneficial effects: HDL cholesterol, insulin sensitivity, and amelioration of inflammation (hsCRP)	[85]
	*kefiranofaciens*	Kefir grains	Anti-colitis	[88]
			Enterohemorrhagic *Escherichia coli* (EHEC) preventing infection and its effects.	[89]
			Antiallergic effect	[90]
*Lactococcus*	*lactis*	Fermented milk	Blood pressure lowering effect	[59]
		Mixture of probiotic bacteria	Atopic dermatitis (eczema) prevention	[91]
*Streptococcus*	*thermophilus*	Yoghurt	Overweight treatment: LDL cholesterol reduction and fibrinogen increase	[92]
		Commercial probiotic	Child obesity and nonalcoholic fatty liver disease (steatohepatitis) treatment	[93]
		Tablets with *Lactobacillus bulgaricus*	Nonalcoholic fatty liver disease treatment	[94]
	*macedonicus*	Commercial probiotic mixture	Overweight beneficial effects: HDL cholesterol, insulin sensitivity and amelioration of inflammation (hsCRP)	[85]
*Leuconostoc*	*lactis*	Fermented milk	Blood pressure regulation (antioxidant and ACE-I activities)	[61]

## Data Availability

Data are contained within the article.
